# Psychological impact of lifestyle-related disease disclosure at general checkup: a prospective cohort study

**DOI:** 10.1186/s12875-015-0272-3

**Published:** 2015-05-14

**Authors:** Tomokazu Tominaga, Masato Matsushima, Takuya Nagata, Akinari Moriya, Takamasa Watanabe, Yuko Nakano, Yoko Hirayama, Yasuki Fujinuma

**Affiliations:** Musashi-Koganei Clinic, Japanese Health and Welfare Co-operative Federation, 1-15-9 Honcho koganeishi, 181-0004 Tokyo, Japan; Division of Clinical Epidemiology, Jikei University School of Medicine, 3-25-8 Nishishimbachi, 105-8461 Minato-ku, Tokyo Japan; Kita-Adachi Seikyo Clinic, Tokyo Hokuto Health Co-operative, 3-1-5 Iriya, 121-0836 Adachi-ku, Tokyo Japan; Japan Small Animal Cancer Center, 2-27-4 Nakatomiminami, 359-0003 Tokorozawa, Saitama Japan; Oji Seikyo Hospital, Tokyo Hokuto Health Co-operative, 3-4-15 Toshima, 114-0003 Kita-ku, Tokyo Japan; Centre for Family Medicine Development, Japanese Health and Welfare Co-operative Federation, 3-22-1 Ukima, 115-0051 Kita-ku, Tokyo Japan; Interprofessional Education Research Center (IPERC), Graduate School of Nursing, Chiba University, 1-8-1 Inohana, Chuo-ku, 260-8670 Chiba City, Chiba Japan

**Keywords:** General checkup, Lifestyle-related disease, Anxiety, Diagnosis

## Abstract

**Background:**

Little is known about psychological impact of disclosing lifestyle-related diseases. Previous studies discussed the long-term psychological impact of disease disclosure, and a significant psychological impact was not observed.

This study clarified the psychological impact on anxiety state of patients when lifestyle-related diseases are disclosed at general checkups for local residents.

In particular, this study evaluated the short-term impact on patients, and how the notification of abnormal values and the disclosure of disease at general checkups affect patients’ subsequent behavioral changes.

**Methods:**

The study design was a prospective cohort study. We compared the anxiety state of participants using a self-administered anxiety assessment scale, State–Trait Anxiety Inventory (STAI), before and after Physician’s explanation of abnormal values in markers of lifestyle-related diseases. The participants were those between the age of 40 and 75 years who underwent general checkups at two primary care facilities. In addition, we assessed the effects on lifestyle habits and the psychological impact caused by general checkup using STAI and a survey on behavioral changes one month after the checkup.

**Results:**

The valid response rate at the survey of the general checkup was 92% (534/578). Of those who showed abnormal levels in markers of lifestyle-related diseases, anxiety was augmented significantly among those who responded that the physician had told them of their diagnosis compared to those who responded that the physician had not told them of their diagnosis (Wilcoxon rank-sum test, P < 0.007). The percentage of patients whose state anxiety scale of STAI increased ≥5 points was 30% in the disease disclosed group (33/111) and 17% in the disease undisclosed group (27/159), respectively. The risk ratio was 1.5 (95% CI: 1.1–2.0). One month after the general checkup, overall anxiety diminished regardless of whether diagnosis of lifestyle-related diseases was disclosed to patients notified of abnormal values. In addition, improvements in daily life behaviors as a result of notification of abnormalities or disclosure of diagnosis at general checkup were not observed.

**Conclusion:**

Even in a general checkup for the general population, disclosing non-critical diseases such as lifestyle-related diseases exacerbated anxiety as a short-term psychological impact.

## Background

In 2008, the new system of mandatory health checkups in Japan called “specific health checkups” was launched to screen for lifestyle-related disease [1,2], the cost of which is covered by local governments. In general, the primary purpose of “health checkups” is to discover disease in early stages, which can lead to improved treatment efficacy. However, according to a meta-analysis conducted by Krogsbøll et al. in 2012 [3], general checkups do not decrease mortality and morbidity, suggesting that their effects still need validation.

From the perspective of psychological impact on individuals, the aforementioned meta-analysis found that only two studies reported relevant results using scales that measure psychological distress, with no significant impact observed [4,5]. However, these previous studies discussed the long-term (1–5 years) psychological effect, and thus the short-term psychological impact remains unknown. Krogsbøll et al. also indicated that over-diagnosis may further augment patients’ anxiety [3]. In addition, previous long-term studies investigated patients who were already receiving care from a medical institution. In other words, these studies were clinic- or hospital-based. In contrast, the new system of general health checkups for local Japanese residents includes those without regular care from medical institutions. Thus, it includes many residents who feel healthy or who do not actively seek medical care on their own. Furthermore, while many studies have assessed the impact of disclosing diseases that affect short-term life prognosis, such as cancer [6-8], most diseases found during general checkups are lifestyle-related diseases, such as dyslipidemia or diabetes, that affect long-term prognosis. Among lifestyle-related disease, only the long-term effects of labeling individuals as having hypertension have been evaluated. In that report, individuals labeled as having hypertension complain of symptoms of depression significantly more often than those who are not [9]; however, the psychological impact of disclosing other lifestyle-related diseases is not clear.

The main objective was to measure anxiety levels (state and trait) before and after consultation when lifestyle-related diseases are disclosed at general checkups for local residents. In addition, we evaluated how the notification of abnormal values and the disclosure of disease at general checkups affect patients’ subsequent behavioral changes.

## Methods

### Study design, setting and participants

This was a prospective cohort study that assessed psychological status over time, before and after a general checkup, using a self-administered survey. The study was conducted at two facilities—a family physician teaching clinic responsible for primary care and a family physician teaching hospital with a 150-bed inpatient facility—in Kita-ku, Tokyo, a district with a large population of elderly residents approximately 15 km north of central Tokyo. Health checkups were conducted intensively between June and October 2011, and self-administered questionnaire assessments were conducted at three time points: before and after the explanation of the checkup results, and one month after the checkup. Instead of an intervention study, the observational study design was chosen because it may be ethically difficult to allocate “no notification” and/or “no disclosure” to participants, and this study focused on the psychological impact from the participants’ perspective.

The system of general health checkups for screening lifestyle-related diseases in Japan, called “specific health checkup”, is as follows: All adults aged 40 years or older who are covered by public health insurance are sent a voucher from the government office of their resident district to receive the health checkups for lifestyle-related diseases, and individuals are to undergo the checkup at a specified medical institution within their district. When individuals visit a medical institution for consultation in this district, they are eligible to receive blood pressure measurement, blood tests, chest X-ray, electrocardiogram, and urine test, free of charge. For the blood test results, values that deviate from pre-determined standard values are automatically identified by a computer, and are output and printed on the results sheet. The general checkup results are explained and guidance is given to the participants by the physician based on the results sheet at the institution where the participants received the general checkup.

Study participants comprised adults aged 40 years or older, but less than 75 years, who received this health checkup between June and October 2011 at one of the two specified facilities, and who did not regularly visit a medical institution and agreed to participate in the study. Those who had difficulty filling out the questionnaire due to reduced visual acuity and those who had been diagnosed with dementia were excluded. The target lifestyle-related diseases in this study were diabetes, dyslipidemia, hypertension, and hyperuricemia.

**Figure 1:**
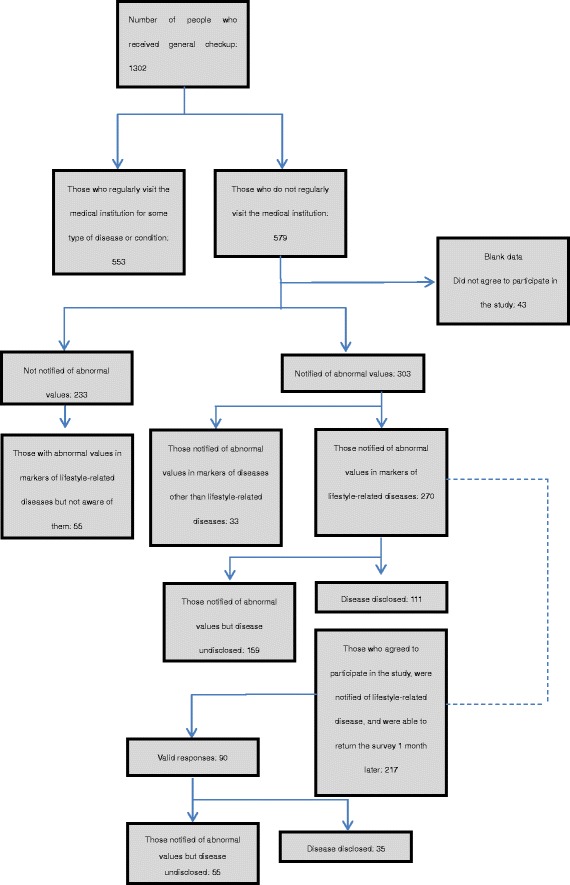
The participant selection process and number of participants.

This study was approved by the Ethics Committee of Ouji Coop Hospital (approval number 41). A bulletin was posted in front of the medical room to provide information about the study, and informed consent was obtained verbally from all participants. The participant selection process is illustrated in Figure 1.

### Measurement variables and evaluation process

The anxiety state of the patient was evaluated using a self-administered questionnaire, the State–Trait Anxiety Inventory (STAI), before the physicians explained the general checkup results. Detailed information about STAI is described in the Instruments section. The participant’s lifestyle habits were assessed using a self-administered questionnaire by determining the stages of behavioral change described by Prochaska [10]. Patients were classified into one of five stages: pre-contemplation, contemplation, preparation, action, or maintenance [11,12]. The stages of behavioral change are assessed for diet, exercise, drinking, smoking, and seeking medical care and are compared before and one month after of the general checkup. We developed a questionnaire that asks participants about their behavioral stages for each of diet, exercise, drinking, smoking, and seeking medical care [13]. Each stage was scored on a scale from 4 to 0 and compared before and one month after the general checkup. If participants’ answer was “Precontemplation stage”, it was scored as 4. The rest of the stages were scored as follows: “Contemplation stage” as 3, “Preparation stage” as 2, “Action stage” as 1, and “Maintenance stage” as 0.

The questions were as follows:

- Diet: What do you think about improving your diet behavior?
- Exercise: What do you think about exercising for 30 min at least twice a week?
- Seeking medical care: What do you think about seeing a doctor regularly?
- Drinking: Appropriate drinking is up to 20 g of alcohol (180 ml of Japanese sake) each day, and the general recommendation is to abstain from drinking alcohol twice a week. What do you think about this habit?
- Smoking: What do you think about quitting smoking?
- For questions about drinking and smoking, since the question is concerned about addictive behavior, we excluded the stage of maintenance according to its definition that the stage extends from six months to an indeterminate period.

Participant’s characteristics (gender, age), screening for depression (two questions) were also assessed using an additional questionnaire.

Immediately after a physician explained the participant’s results from the health checkups, his/her anxiety state was evaluated again using STAI. An additional questionnaire was also administered to ask whether the physician had told the participants of any abnormal results or had diagnosed a lifestyle-related disease, and if so, what the diagnosis was.

The purpose of this study was masked from the physicians who explained the results of general checkups to participants, and they were given instructions to approach the participants as they normally would. The automatic assessment criteria for abnormal levels were determined according to the International Diabetes Federation (IDF) standard from 2005 and Third Report of the National Cholesterol Education Program Adult Treatment Panel III (NCEP-ATPIII) standards [14], and were as follows: ≥130/85 mmHg for hypertension, ≥150 mg/dL for triglycerides, <40 mg/dL (men) and <50 mg/dL (women) for HDL-c, and ≥100 mg/dL for plasma glucose. The abnormal level for uric acid for both men and women was >7.0 mg/dL, which is the upper normal limit commonly used in Japan [15]. HbA1c was also considered as a marker for diabetes, and HbA1c ≥6.5% (NGSP) was determined as abnormal [16,17]. In addition, considering the possibility that the level of abnormality in markers is likely to have an effect on the physician’s explanation to a participant, we also conducted analysis for only those with mild abnormality (mild group) since the farther the result is from the reference value, the more confidently and assertively a physician would explain the result and diagnosis. Those with mild abnormality were not in the stage that would generally require immediate treatment at medical consultations in Japan, but had borderline high values that require attention according to NCEP-ATP III and AHA guidelines [11,18,19]. The following values were used as criteria to determine mild abnormalities: systolic blood pressure of 140–159 mm Hg and diastolic blood pressure of 90–99 mm Hg for hypertension, triglycerides of 150–199 mg/dL and LDL of 150–159 mg/dL for dyslipidemia, and HbA1c (NGSP) of 6.5–7.0% for diabetes [16,17]. In those who were pointed out to have a high marker for uric acid, patient recollections were usually concerned with hyperuricemia treatment that would usually apply to patients with a history of gout; therefore, uric acid levels were not used as a criterion of mild abnormality.

The collection staff monitored participants as they filled out the self-administered questionnaire before and after the general checkup, and the questionnaires were recovered from all participants in front of the medical room after the checkup. One month after the general checkup results were explained to a study participant at a physician’s consultation, a questionnaire was mailed to those who had abnormal levels in markers of lifestyle-related disease. The replies are sorted into those who were notified of abnormalities in markers or who were notified of a diagnosis of lifestyle-related disease. This questionnaire included STAI, as well as additional questions on stages of behavioral changes in diet, exercise, drinking, smoking, and seeking medical care, and whether or not their lifestyle habits had improved. Explanation to the participants was given as “the questionnaire is asking whether your lifestyle has changed after the health checkup and about anxiety change before and after the health checkup. The questionnaire strictly focuses on the health checkup, and answers would not affect any action resulting from your regular consultation.”

The questionnaire was filled out by the study participants and returned by mail. Self-administered questionnaires that contained blanks or were difficult to evaluate were treated as missing data and were excluded from the analysis.

In the present study, patients were directly asked whether they were notified of abnormalities by the physician, and whether the diagnosis was disclosed to them. The reason why the physicians were not surveyed about their decision for participants’ results was that conducting a survey for the physicians who explained the results of general checkup including abnormalities and disease disclosure would affect their clinical practice style during the study period, potentially creating a bias. As this study focuses on a natural process of doctor–patient interaction, the purpose of this study was obliged to be concealed to doctors. In addition, the purpose of the present study was not to investigate the intervention effect by disclosing the diagnosis to patients but to focus on the psychological impact of general checkup results on patients based on the patient’s perception. In addition, it may be ethically difficult to allocate “no notification” and/or “no disclosure” as physicians’ behavior to participants. Therefore, we considered that the impact of disease disclosure should be evaluated from the perspective of the patients first.

### Instruments

STAI, developed in 1966 using Spielberger’s theory, is a 40-item questionnaire that measures both state anxiety and trait anxiety. State anxiety is an anxiety state induced in a short period of time by situations that are perceived as harmful. Trait anxiety is an anxiety state caused by one’s innate personality. In the present study, both state anxiety and trait anxiety were assessed by state-Trait Anxiety Inventory (Japanes version) [20,21] to determine whether the anxiety state was greatly affected by the patient’s innate personality or induced by purely being notified of a disease condition.

### Size of study

In order to test the hypothesis that anxiety was induced by receiving notifications of an abnormality or disease at a general checkup, the number of samples required to conduct the study was determined in advance. Each question in STAI is rated on one of the following 4-point scales (listed in increasing point value): “very much so, moderately so, somewhat, not at all” or “almost never, sometimes, often, almost always”. Scores of 1–4 points are added, and the total scores range from 20 to 80. The total score is classified into the following five levels of anxiety: very high, high, normal, low, and very low. There are approximately 10 points between each of these levels. When the mean change in the STAI score upon receiving notification at the general checkup was assumed to be 5 points and standard deviation (SD) was assumed to be 15, the number of samples statistically required for the study became 190 for one group (α = 0.05, β = 0.10). In order to compare the groups of people whose anxiety was induced versus not induced, the number of samples required became 380. Therefore, the target sample number was set to approximately 400.

### Analysis and statistical methods

The following two aspects were assessed for: 1) whether the participant’s anxiety scale STAI score is affected; and 2) whether there are behavioral changes in daily lifestyle.

1. Participants were divided into three groups: those not notified of any abnormalities in markers of lifestyle-related disease through automatic assessment (hereinafter “no abnormality group”), those notified of abnormalities and were told of the disease diagnosis by the physician (“disease disclosed group”), and those notified of abnormalities but were not told of the disease by the physician (“notified of abnormality/disease undisclosed group”). Whether or not the changes in state of anxiety are different between each of the two groups was determined using Student’s *t* test for parametric data and the Wilcoxon rank-sum test for nonparametric data. Bonferroni correction for multiple comparisons was applied in this case. The Shapiro–Wilk test was used to determine whether the data were parametric or nonparametric. As shown in Figure 1, those who responded that they were notified of abnormalities in diseases other than lifestyle-related diseases were excluded from the analysis. For changes in state of anxiety, in order to evaluate the independent impact of disease diagnosis disclosure, logistic regression analysis adjusting for gender, age, depressed mood, loss of interest, trait anxiety, diabetes, hyperlipidemia, hypertension, and hyperuricemia as covariates was conducted [22]. Logistic regression analysis was similarly conducted in a group of participants limited to those with mild abnormalities in lifestyle-related disease markers. The anxiety assessment scale STAI was used in the present study, and its results were classified into five levels with each level approximately ten points apart. A higher total score indicates higher anxiety, with the five levels having anxiety levels of very high, high, normal, low, and very low. In the logistic regression analysis, we constructed two models, one using the five levels that are approximately ten points apart in each level and another using half-levels that are approximately five points apart in each level as outcome development of clinically significant changes in state anxiety.
2. Changes in lifestyle habits one month after the explanation of general checkup results between the notified of abnormality/disease undisclosed group and disease disclosed group were compared using the Wilcoxon rank-sum test. In addition, Spearman’s correlation coefficients were used to elucidate the association between change of state anxiety before and just after the checkup, and changes of behavioral stages one month after the checkup.

All statistical analyses were performed using STATA/SE version 10.1. P < 0.05 was considered to be statistically significant [23].

## Results

There were 449 participants from the first site (clinic) and 853 from the second site (hospital), and 242 out of the 449, and 337 out of the 853 participants did not regularly visit a medical institution and agreed to participate in this study. The characteristics of participants in this prospective cohort study are shown in Table 1. The male-to-female ratio was 182:321. The mean age (±SD) was 62 ± 9 years. Many participants were around retirement age, which is around 60–65 years in Japan.

**Table 1:** **Characteristics of patients**

	***Total No.***	***abnormality group***	***Notified abnormality/Disease undisclosed group***	***Disease disclosed group***
*Number of patients*	***503***	***233***	***159***	***111***
*Gender (male/female)*	***182/321***	***68/165***	***66/93***	***48/63***
*Age (Mean age ± SD)*	***62.2 ± 9.1***	***61.9 ± 9.4***	***62.0 ± 9.0***	***63.3 ± 8.9***
Number of patients by disease*				
*Hypertension*	***57 (11%)***	***17 (7%)***	***24 (15%)***	***16 (14%)***
*Dyslipidemia*	***169 (34%)***	***43 (18%)***	***71 (45%)***	***55 (50%)***
*Diabetes*	***23 (4%)***	***1 (0.4%)***	***9 (6%)***	***13 (12%)***
*Hyperuricemia*	***35 (7%)***	***7 (3%)***	***15 (9%)***	***13 (12%)***
*With or without depression tendency*				
*Depression tendency*	***106 (21%)***	***38 (16%)***	***35 (22%)***	***33 (30%)***

*One patient may be afflicted by more than one disease. For “with or without depression tendency”, patients who answered yes to either or both of the two questions regarding depression (loss of interest, depressed mood) were considered to have a depression tendency.

*SD: standard deviation.*

**Figure 2:**
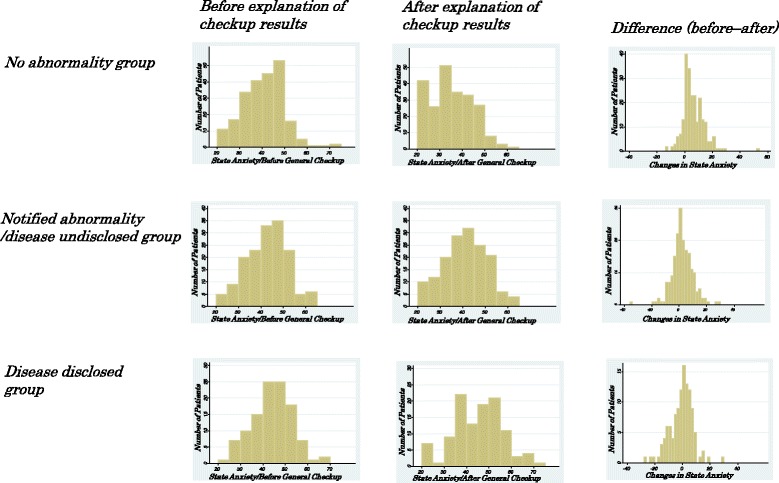
Comparison of changes in state anxiety before and after general checkup (no abnormality, notified of abnormality/disease undisclosed, and disease disclosed groups). The x-axis of the histograms in the left and middle columns represents the state anxiety score on the STAI. The histograms in the right column indicate changes between before and after general checkup results were explained by the physician. The graphs in the right column are skewed to the left with increased anxiety and skewed to the right with decreased anxiety.

Approximately 60% of the participants had abnormal values in markers of lifestyle-related disease, and diseases were disclosed to less than half of these participants. As shown in Figure 2, state anxiety before and after explanation of the results by the physician was compared in no abnormality, notified of abnormality /disease undisclosed, and disease disclosed groups. In no abnormality and notified of abnormality/disease undisclosed groups, the state anxiety decreased after participants received an explanation of the results. In contrast, state anxiety was exacerbated in the disease disclosed group.

Statistically, state anxiety was also augmented significantly in the disease disclosed group compared to the notified of abnormality/disease undisclosed group (Wilcoxon rank-sum test, P < 0.007). Moreover, in both comparisons between the no abnormality group and notified of abnormality/disease undisclosed group, and between the no abnormality group and disease disclosed group, the disease undisclosed and disease disclosed groups showed a significantly augmented state anxiety compared to the no abnormality group (Wilcoxon rank-sum test, P < 0.001).

The percentage of patients whose state anxiety scale of STAI (maximum score: 80) increased ≥5 points was 30% in the disease disclosed group (33/111) and 17% in the disease undisclosed group (27/159), respectively. The risk ratio was 1.5 (95% CI: 1.1–2.0). After adjusting for gender, age, depressed mood, loss of interest, trait anxiety, diabetes, hyperlipidemia, hypertension, and hyperuricemia as covariates in a logistic regression model (Table 2), the odds ratio of state anxiety increasing by ≥5 points due to disease disclosure was 2.1 (95% CI: 1.1–4.0). Similarly, the odds ratio of state anxiety increasing by ≥10 points was 3.0 (95% CI: 1.2–7.0). This indicated that disease disclosure augments state anxiety even after taking into consideration trait anxiety, which indicates anxiety tendency due to innate personality. In addition, logistic analysis adjusted for covariates was similarly conducted in the mild group, and showed that the risk of state anxiety increasing by ≥5 points was significantly elevated in the disease disclosed group (odds ratio 3.1, 95% CI: 1.2–8).

**Table 2:** **Analysis of factors that affect changes in anxiety**

	**STAI state anxiety increased by 5 points**	**STAI state anxiety increased by 10 points**
***Unadjusted***		
	*Risk Ratio (95% CI)*	*Risk Ratio (95% CI)*
*With disease disclosure*	*1.4 (1.10–1.97)*	*1.60 (1.16–2.22)*
***Adjusted***		
	*Odds Ratio (95% CI)*	*Odds Ratio (95% CI)*
*With disease disclosure*	*2.09 (1.10–3.99)*	*2.97 (1.25–7.02)*
*Gender*	*1.49 (0.74–3.01)*	*1.58 (0.62–4.04)*
*Age*	*0.98 (0.94–1.01)*	*0.97 (0.93–1.02)*
*Depressed mood*	*0.47 (0.14–1.48)*	*0.67 (0.14–3.27)*
*Loss of interest*	*0.88 (0.22–3.57)*	*1.51 (0.23–9.88)*
*Hypertension*	*1.28 (0.54–3.02)*	*1.47 (0.48–4.49)*
*Dyslipidemia*	*1.05 (0.55–2.01)*	*1.35 (0.57–3.20)*
*Diabetes*	*1.52 (0.43–5.37)*	*1.99 (0.45–8.80)*
*Hyperuricemia*	*0.66 (0.20–2.17)*	*0.87 (0.21–3.51)*
*Trait anxiety*	*0.97 (0.93–1.01)*	*0.93 (0.88–0.98)*

*STAI: State-Trait Anxiety Inventory.*

*CI: Confidence interval.*

Table 2 Shows the comparison of logistic analysis results adjusted for covariates between the abnormality notified/disease undisclosed and disease disclosed groups in order to elucidate whether disclosing disease increases state anxiety.

The mailed survey recovery rate one month after the general checkup was 42% (90/217). State anxiety significantly decreased in both those who are notified of abnormal levels of lifestyle-related disease markers or whose disease was disclosed at one month after the general checkup compared to right after the checkup (paired *t* test, P < 0.015, P < 0.006, respectively). State anxiety at one month after the checkup was not significantly different between the notified of abnormality/disease undisclosed group and disease disclosed group (Student’s *t* test, P < 0.85).

Between those who responded to the mailed questionnaire at one month after and those who did not, neither the change of state anxiety nor trait anxiety before and just after the general checkup showed any significant difference (Wilcoxon rank sum, P < 0.11).

**Figure 3:**
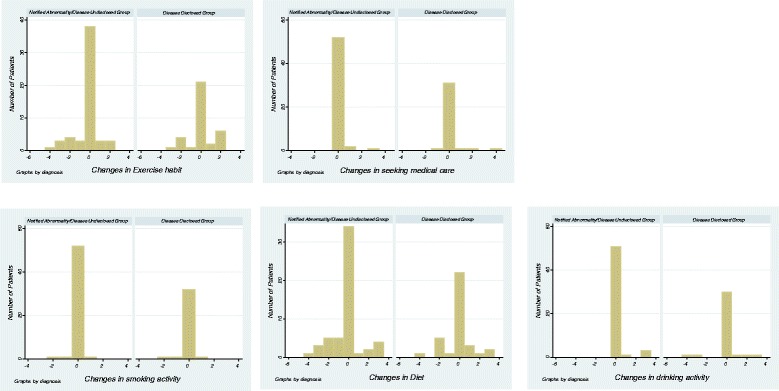
Improvements in lifestyle habits one month after general checkup. The y-axis of the graphs represents stages of behavioral changes in each of the lifestyle habits. If the stages of behavioral changes were not different one month after the general checkup compared to before the general checkup, the value was 0. If the behavioral changes improved by one stage, then the value was 1.

The percentage of participants who responded that there was an improvement in their own lifestyle habits one month after the general checkup was 40.0% for the notified of abnormality/disease undisclosed group and 37.1% for the disease disclosed group, demonstrating that significant differences were not observed between the two groups (Wilcoxon rank-sum test). Figure 3 shows the comparison between before and one month after the general checkup on whether lifestyle habits such as diet, drinking, exercise, smoking, and seeking medical care actually improved. Significant improvements were not observed in any of these variables. There was no statistically significant difference in Spearman’s correlation coefficients between the change of state anxiety and changes of behavioral stages.

## Discussion

The patient’s anxiety state is augmented more by disclosure of disease “labeling”, or diagnosis from the physician at a general checkup than by a simple notification of abnormal values. Excluding the effects of potential confounding variables such as anxiety traits or depressive states associated with personality and types of lifestyle-related diseases also led to similar results. Additionally, even with mild deviations in the abnormal test values, disease diagnosis disclosure elicited an anxiety state in patients. Despite these results, improvements in daily life behavior due to the perception of abnormality notification or diagnosis disclosure were not observed one month after the general checkup. The results of the present study showed that, even in a general checkup for the general population, disclosing non-critical diseases such as lifestyle-related diseases exacerbated anxiety as a short-term psychological impact.

Anxiety state is thought to be affected by many factors. In particular, anxiety state related to personality, namely trait anxiety, is an important variable that should be adjusted as a confounding factor. Therefore, in order to elucidate the independent impact of disease diagnosis disclosure on anxiety state, adjustments for trait anxiety, gender, age, depressed mood, loss of interest, kinds of diseases were conducted as covariates. The results indicated that diagnosis disclosure augmented the patients’ anxiety state independent of the effects from these possible covariates. However, other factors that affect the patient–physician relationship, such as duration of consultation, the specific method in which the physician discloses information, and the amount and content of the explanation aside from disease disclosure, were not assessed in the present study, and these may have affected the results. In particular, from the perspective of communication between patient and physician, one of the points that must be considered is that a physician may be more apt to disclose the disease to patients when there is a greater deviation from normal test values. However, even when we analyzed participants whose test results were in the “mild” range, anxiety was augmented due to disease disclosure. Therefore, disclosing the diagnosis was found to exacerbate anxiety, even if there was only mild abnormality in lifestyle-related disease markers.

This study has considered the differences in the comprehension of one’s own health state between local residents and patients registered at a clinic. This has revealed how local resident’s notification of abnormal values for lifestyle-related disease markers and diagnosis disclosure truly affect those who consider themselves healthy or believe they are not ill. Although we investigated the disclosure of lifestyle-related diseases that are not directly life threatening in the early stages, there was a negative psychological impact on local residents who must be more sensitive to labeling than patients regularly visiting a clinic, similar to the psychological impact of patient labeling by cancer diagnosis [6-8]. This may be attributed in part to physicians who are not aware of the psychological impact of the diagnosis of lifestyle-related diseases on patients, especially on local residents. In the future, the explanation of lifestyle-related disease results while considering the psychological impact on patients, similar to disclosing a cancer diagnosis, will be necessary.

It was also suggested that lifestyle habits might not be improved one month after the general checkup despite a low follow-up rate. From the results of behavioral changes one month after the checkup, improvements in daily life behaviors were not observed despite patients’ perception that they were notified of abnormal values or disclosed a diagnosis at general checkup. There was no indication in the present study that patients with higher anxiety score made greater attempts to improve their behavior. Moreover, at one month after, overall anxiety had decreased in patients who were notified of abnormalities regardless of the disclosure of lifestyle-related disease by the physician, demonstrating that anxiety merely increased temporarily immediately after the general checkup. This result most likely indicates that, depending on the method, notification of abnormal values and diagnosis disclosure at general checkups do not necessarily elicit improvements in behavior, but rather temporarily augments psychological anxiety. One reason for this is that physicians may be uniformly disclosing the diagnosis regardless of the severity. In other words, this may be augmenting anxiety in those with mild illnesses while greater intervention may not be performed in those with severe illnesses. If anxiety alone is augmented without improvements in lifestyle habits due to general checkup results, then this deviates from the idea of conducting general checkups with the primary purpose to discover lifestyle-related diseases early and prevent them. Considering the results of this study, further studies to evaluate the way of delivering the information to motivate people should be done soon. It is also an immensely important issue that only a few participants sought to improve their health one month after the health checkup. This issue has to be further examined as well.

There are several limitations to this study. The questionnaire response rate by mail at one month after general checkup was low. Thus, it may be difficult to draw definitive conclusions on behavioral changes after the general checkup. Among non-responders to the mailed questionnaire, however, the change of anxiety between before and just after the general checkup was not significantly different from that among responders, which means responders had no specific characteristics on anxiety change against labeling. Moreover, even if non-responders might be less motivated to modify their lifestyle than those who responded, labeling could have made less of an impact than observed. In addition, the details of the study population, specifically whether it deviated from the entire resident population, could not be analyzed. Although the specific health checkup is mandatory, the percentage of those who receive it is not necessarily 100%; thus, the study population may comprise many people who are particularly interested in their own health. Moreover, because the study investigated those who are able to receive checkups on weekdays, employees who have difficulty receiving checkups on weekdays are thought to be absent from the study population. This was considered the reason why more women received checkups. However, at the very least, there were no significant differences in the changes in anxiety between men and women. Another limitation is that the lifestyle behavior was evaluated by a self-rating questionnaire of behavioral stages, and not by the actual behaviors or specific indices such as calorie consumption or physical activity.

Future investigations should be conducted on the method in which general checkup results are explained, keeping in mind that the manner in which lifestyle-related diseases are disclosed or in which patients are notified of abnormal values may augment patients’ psychological anxiety.

## Conclusion

Even in a general checkup for the general population, disclosing non-critical diseases such as lifestyle-related diseases exacerbated anxiety as a short-term psychological impact. In the future, the explanation of lifestyle-related disease results while considering the psychological impact on patients will be necessary.
